# A note on age differences in mood-congruent vs. mood-incongruent emotion processing in faces

**DOI:** 10.3389/fpsyg.2014.00635

**Published:** 2014-06-26

**Authors:** Manuel C. Voelkle, Natalie C. Ebner, Ulman Lindenberger, Michaela Riediger

**Affiliations:** ^1^Center for Lifespan Psychology, Max Planck Institute for Human DevelopmentBerlin, Germany; ^2^Department of Psychology, University of FloridaGainesville, FL, USA

**Keywords:** emotion perception, mood-(in)congruent information processing, mood regulation, faces, crossed random effects analysis

## Abstract

This article addresses four interrelated research questions: (1) Does experienced mood affect emotion perception in faces and is this perception mood-congruent or mood-incongruent?(2) Are there age-group differences in the interplay between experienced mood and emotion perception? (3) Does emotion perception in faces change as a function of the temporal sequence of study sessions and stimuli presentation, and (4) does emotion perception in faces serve a mood-regulatory function? One hundred fifty-four adults of three different age groups (younger: 20–31 years; middle-aged: 44–55 years; older adults: 70–81 years) were asked to provide multidimensional emotion ratings of a total of 1026 face pictures of younger, middle-aged, and older men and women, each displaying six different prototypical (primary) emotional expressions. By analyzing the likelihood of ascribing an additional emotional expression to a face whose primary emotion had been correctly recognized, the multidimensional rating approach permits the study of emotion perception while controlling for emotion recognition. Following up on previous research on mood responses to recurring unpleasant situations using the same dataset (Voelkle et al., 2013), crossed random effects analyses supported a mood-congruent relationship between experienced mood and perceived emotions in faces. In particular older adults were more likely to perceive happiness in faces when being in a positive mood and less likely to do so when being in a negative mood. This did not apply to younger adults. Temporal sequence of study sessions and stimuli presentation had a strong effect on the likelihood of ascribing an additional emotional expression. In contrast to previous findings, however, there was neither evidence for a change from mood-congruent to mood-incongruent responses over time nor evidence for a mood-regulatory effect.

## Introduction

How does the way we feel influence the perception of the world around us, and how does this perception in turn affect our own feelings? As innocuous as it may seem, this question constitutes one of the most fundamental research objectives in psychology, ranging from basic research on attention and perception (e.g., Becker and Leinenger, 2011; Hunter et al., 2011) to research in clinical psychology and psychiatry (e.g., Elliott et al., 2002; Eizenman et al., 2003; Rinck et al., 2003; Stuhrmann et al., 2013). For example, cognitive theories of anxiety and depression suggest attentional and memory biases of patients suffering from anxiety or depression toward threatening, respectively dysphoric, stimuli, which in turn contributes to the maintenance or aggravation of the disorder (Clark et al., 1999; Beevers and Carver, 2003; Koster et al., 2010). In particular the question of mood-congruent vs. mood-incongruent information processing, its determinants and consequences, has sparked a lot of research. The present paper contributes to this literature by investigating the relationship between experienced mood and the perception of emotional expression in faces, which has been shown to be important for individuals' social interactions (e.g., Baron-Cohen et al., 2000. Special emphasis will be put on age-related differences in mood-(in)congruent information processing, the role of the temporal sequence of study sessions and stimuli presentation, and the question whether mood may not only affect emotion perception, but whether emotion perception may also serve a mood-regulatory function.

As will be outlined in the following, a number of different theoretical models have been proposed to explain the—in parts conflicting—empirical findings on mood-congruent vs. mood-incongruent information processing:

### Mood-congruent information processing

There exists ample evidence that information is often processed in a *mood-congruent* manner. For example, people in a positive mood are more likely to recall positive memories (Bower, 1981, 1991; Mayer et al., 1995), and report to be more satisfied with their lives (Schwarz and Clore, 1983). Mood congruency effects were also observed for emotion perception in faces. The pattern of findings, however, is somewhat mixed. For example, Coupland et al. (2004) demonstrated that low positive affect (anhedonia) decreased the identification of happy expressions, while negative affect increased the identification of disgust. Contrary to expectations, however, there was no increase in anger identification related to negative affect. Furthermore, Suzuki et al. (2007) found partial support for a mood-congruency effect by observing a positive correlation between negative affect and recognition of sadness. However, this effect did not generalize to other negative emotions. In addition, age-related decrease in sadness recognition was linked to an age-related decrease in negative affect. For similar findings on the relation between age-related decline in the perceived intensity of emotions in faces and age-related decrease in anxiety and depression see Phillips and Allen (2004).

At a more general level, the mood-congruency effect has been explained in terms of Bower's (1981) *associative network theory*. This theory refers to the idea that emotions serve as memory units and that activation of such a unit not only “aids retrieval of events associated with it [but…] also primes emotional themata for use in free association, fantasies, and perceptual categorization” (Bower, 1981, p. 129). The effect has also been explained in terms of *mood as information* (Schwarz and Clore, 2003, p. 296), that is, the idea that mood may serve an informational function and may help in directing attention to possible sources of feelings (Wyer and Carlston, 1979; Schwarz and Clore, 1983, 2003). Although both approaches suggest mood-congruent information processing, the proposed mechanisms differ. According to the *associative network theory*, mood influences information processing *indirectly* by priming the encoding, retrieval, and use of information, for example by selectively attending to “activated” mood-congruent details in the environment, by selectively encoding information into a network of primed associations, or by selectively retrieving mood-congruent information. According to the *mood-as-information account*, mood influences information processing *directly*. By (implicitly) asking themselves “… how do I feel about this? [… people…] misread their current feelings as a response to the object of judgment, resulting in more favorable evaluations under positive rather than negative moods, unless their informational value is discredited” (Schwarz and Clore, 2003, p. 299).

In an attempt to integrate these seemingly contradictory explanations, Forgas (1995) proposed the *affect infusion model (AIM)*, which states the more general preconditions for mood-congruency effects in judgmental processes. According to the AIM, affect-priming, in the sense of Bower's (1981) associative network theory, is most likely to occur during *substantive processing*, that is, in situations with complex, atypical, and/or personally relevant targets. For example, when being in a happy mood, people evaluate others more favorably than when being in a sad mood, in particular when judging unusual, atypical, persons (Forgas, 1992).

In contrast, mood-as-information (Schwarz and Clore, 1983) is the major affect infusion mechanism during *heuristic processing*, that is, in situations involving typical targets of low personal relevance and/or in situations with limited processing capacity (e.g., due to time pressure or information overload; Forgas, 1995).

### Mood-incongruent information processing as a mood-regulatory function

In addition to the various findings on mood-congruency effects, a number of studies suggested that information may also be processed in a *mood-incongruent* manner (Morris and Reilly, 1987; Matt et al., 1992; Erber and Erber, 1994; Sedikides, 1994; Forgas and Ciarrochi, 2002; Isaacowitz et al., 2008, 2009b). For example, in two recent articles Isaacowitz and colleagues (Isaacowitz et al., 2008, 2009b) showed that older adults gazed toward positively valenced facial stimuli when in a bad mood. In contrast, a mood-congruency effect was observed in younger adults, in that they were more likely to look at positively valenced faces when in a good mood and more likely to look at negatively valenced faces when in a bad mood. Based on the observed age-differential relationship between mood and gazing pattern, Isaacowitz et al. (2008) concluded that “in older adults, gaze does not reflect mood, but rather is used to regulate it” (2008; p. 848). This interpretation is in line with socioemotional selectivity theory, which postulates that older adults—because of a shrinking time horizon—shift their motivational priorities toward emotion regulation (Carstensen et al., 1999; Carstensen, 2006). Furthermore, given that most studies on mood-(in)congruent information processing used college-student populations, this finding cautions generalizations to other populations and underscores the importance of studying different age groups.

Building upon the *first, congruency; then, incongruency* hypothesis postulated by Sedikides (1994, p. 163), Forgas and Ciarrochi (2002) showed, in a series of three experiments, that after an initial mood-congruency effect, people in a sad mood were more likely to generate positive person descriptions, positive personality trait adjectives, as well as positive self-descriptions. These findings were interpreted in terms of a spontaneous, homeostatic, mood management mechanism “that limit[s] affect congruence and thus allow[s] people to control and calibrate their mood states by selectively accessing more affect-incongruent responses over time” (Forgas and Ciarrochi, 2002, p. 337). Thus, the temporal sequence of information processing seems to play a crucial role in the interplay between experienced mood and information processing. This may also apply to the processing of emotional expressions in faces as investigated in the present study.

What the studies by Isaacowitz and colleagues and Forgas and colleagues have in common is that differences in mood-congruent vs. mood-incongruent information processing are explained in terms of mood regulation. That is, by focusing on stimuli of a certain valence, people attempt to manage their mood (e.g., they up-regulate their mood when previously in a bad mood). In contrast to work on mood-congruent attentional and memory biases for negatively valenced material, the mechanisms underlying a mood-incongruent bias toward positively valenced stimuli have been less clearly spelled out. However, given the functional relevance of mood-congruent information processing for dysphoria and depression (e.g., Clark et al., 1999; Beevers and Carver, 2003; Koster et al., 2010), it seems reasonable to assume that focusing on positively valenced stimuli when in a bad mood may help to counteract this effect. The underlying mechanism may either constitute a rather spontaneous, homeostatic mood management (Forgas and Ciarrochi, 2002) or active mood regulation.

While Isaacowitz and colleagues explained the mood-congruency vs. mood-incongruency effect *in terms of age* (“mood-congruent gaze in younger adults, positive gaze in older adults”, Isaacowitz et al., 2008, p. 848), Forgas and Ciarrochi explained the effect *in terms of elapsed processing time* (“initially mood-congruent responses tend to be automatically corrected and reversed over time,” Forgas and Ciarrochi, 2002, p. 344; see also Sedikides, 1994). Such mood-incongruent information processing is also in line with the AIM, which postulates that mood-congruency effects will be eliminated, or reversed, if a person is influenced by a strong motivational component, such as to improve mood when being in a bad mood (i.e., motivated processing; Erber and Erber, 1994; Forgas, 1995). In the absence of a strong motivational component, the AIM proposes the *direct access strategy* as another type of a low affect infusion strategy, that is, an information processing strategy that is unlikely to result in a mood-congruency effect. “Direct access processing is most likely when the target is well known or familiar and has highly prototypical features that cue an already-stored and available judgment, the judge is not personally involved, and there are no strong cognitive, affective, motivational, or situational forces mandating more elaborate processing” (Forgas, 1995, p. 46). We will get back to this strategy in the discussion. For a more detailed description of the AIM, and the four alternative processing strategies related to low affect infusion (motivated processing and direct access strategy) and high affect infusion (heuristic and substantive processing), we refer the reader to Forgas (1995).

To summarize, current research has provided ample support for mood-congruent, but also mood-incongruent, information processing. The AIM provides a general framework that predicts the degree of mood-(in)congruency in information processing (Forgas, 1995). According to this model, mood-congruency effects are most likely under *substantive processing* or *heuristic processing* which is in line with Bower's (1981) associative network theory, and Schwarz and Clore's (1983) theory of mood-as-information, respectively. In contrast, mood-congruency is least likely in case of *motivated processing* or the *direct access* strategy. Especially when in a bad mood, people may be motivated to change this state. As suggested by Isaacowitz et al. (2008), this motivation may be particularly strong in older adults. Furthermore, Forgas and Ciarrochi (2002) observed a shift from mood-congruent to mood-incongruent information processing, pointing to the role of homeostatic cognitive strategies in affect regulation. However, when explaining mood-incongruent information processing in terms of mood regulation, the crucial—and often untested—question is how effective is it in changing peoples' mood? In a recent eye-tracking study, Isaacowitz et al. (2009a,b) showed that older adults with good cognitive functioning showed less mood decline throughout the study when gazing toward positively valenced faces. This provides initial support for the notion that in some people mood-incongruent information processing may serve a mood-regulatory function.

Overall, the research reviewed so far indicates that effects of experienced mood on information processing are by no means simple, but influenced by multiple factors. With few exceptions (e.g., Mayer et al., 1995), most studies on mood-(in)congruent information processing involved active mood induction. Little is known about the extent to which these findings generalize to naturally occurring mood. Moreover, in real-life situations the various mechanisms underlying mood-(in)congruent information processing may work simultaneously and are likely to influence each other. For example, being in a moderately gloomy mood may prime the associative network toward the perception of negatively valenced features in the environment. This in turn may be perceived as more negative because of one's gloomy mood (mood-as-information). At the same time, this may increase the motivation to improve one's mood by selectively attending to positively valenced features in the environment, possibly eliminating or even reversing a mood-congruency effect. At present it is unclear which of these processes will prevail under less extreme conditions of natural mood, rather than experimentally induced mood.

## This study

The purpose of the present study was to link naturally occurring fluctuations in mood to the perception of emotions in faces in order to provide new insights into mood-congruent vs. mood-incongruent information processing. To this end we (1) examined whether natural mood affects emotion perception in faces and to what extent this perception was mood-congruent or mood-incongruent. We (2) tested for age group differences in the interplay between emotion perception and experienced mood, and (3) investigated the role of temporal sequence in emotion processing. Finally, we (4) examined the extent to which emotion perception in faces may serve a mood-regulatory function.

Based on previous findings reviewed above, and independent of age, we expected mood-congruent processing of emotional expressions in faces—operationalized as a higher likelihood of perceiving a positively valenced emotional expression when in a good mood, and a negatively valenced emotional expression when in a bad mood (*Hypothesis 1*). In line with prior research, but competing with Hypothesis 1, we expected older adults to have a higher likelihood of perceiving positive emotions in facial expressions when in a bad mood (*Hypothesis 2*). In addition, we expected a shift from mood-congruent to mood-incongruent information processing as a function of processing time (*Hypothesis 3*). Based on prior research, we expected a positive relationship between the likelihood of perceiving positively valenced emotions in faces and subsequent improvements in mood (*Hypothesis 4*), supporting the notion of a mood-regulatory function of emotion perception.

To test these hypotheses, we asked young, middle-aged, and older adults to indicate the amount of happiness, sadness, fear, disgust, anger, and neutrality they perceived in photographs of faces displaying prototypical happy, sad, fearful, disgusted, angry, or neutral facial expressions. Of importance, using a multidimensional rating approach, participants had rated all six emotions for each prototypical facial expression. For example, although a face may have been correctly recognized as displaying anger (prototypical primary expression), participants could indicate that they also perceived some sadness, or any of the other emotion(s), in the same face. In terms of the AIM, recognizing a prototypical facial expression is likely the result of either a direct access or motivational processing strategy and thus unlikely to infuse affect (Forgas, 1995). In addition, individuals differ in their ability to recognize prototypical facial expressions, with age as an important predictor of these differences (see Ruffman et al., 2008, for an overview)^1^. For these reasons we were *not* interested in emotion recognition, but in the likelihood of ascribing an *additional* emotional expression to a face, whose primary emotion had already been correctly recognized. This procedure controls^2^ for differences in emotion recognition and maximizes the likelihood of affect infusion (i.e., a mood-congruency or mood-incongruency effect). Throughout the remainder of this paper, the term e*motion perception* thus refers to the perception of additional emotional expressions in a face (other than the primary expression), once its primary emotional expression had been correctly recognized (see Methods section for details).

The present article follows up on previous work with the same dataset in which we investigated anticipatory and reactive mood changes throughout the course of the study (Voelkle et al., 2013), age-of-perceiver and age-of-rater effects on multidimensional emotion perception (Riediger et al., 2011), and accuracy and bias in age estimation (Voelkle et al., 2012). Until now, however, we never linked participants' mood to the perception of emotion in others (faces), which is the primary aim of the present study.

## Methods

### Participants

One hundred fifty-four adults of three different age groups (*n* = 52 younger adults: 20–31 years; *n* = 51 middle-aged adults: 44–55 years; *n* = 51 older adults: 70–81 years) participated in the study. All of the 76 women and 78 men were Caucasian and German-speaking. Self-reported physical functioning was good, and visual-processing speed, as assessed by Wechsler's (1981) digit symbol substitution test, was comparable to typical performance levels in these age groups. For details about the demographic composition of the sample, see Ebner et al. (2010).

### Procedure

**Figure 1:**
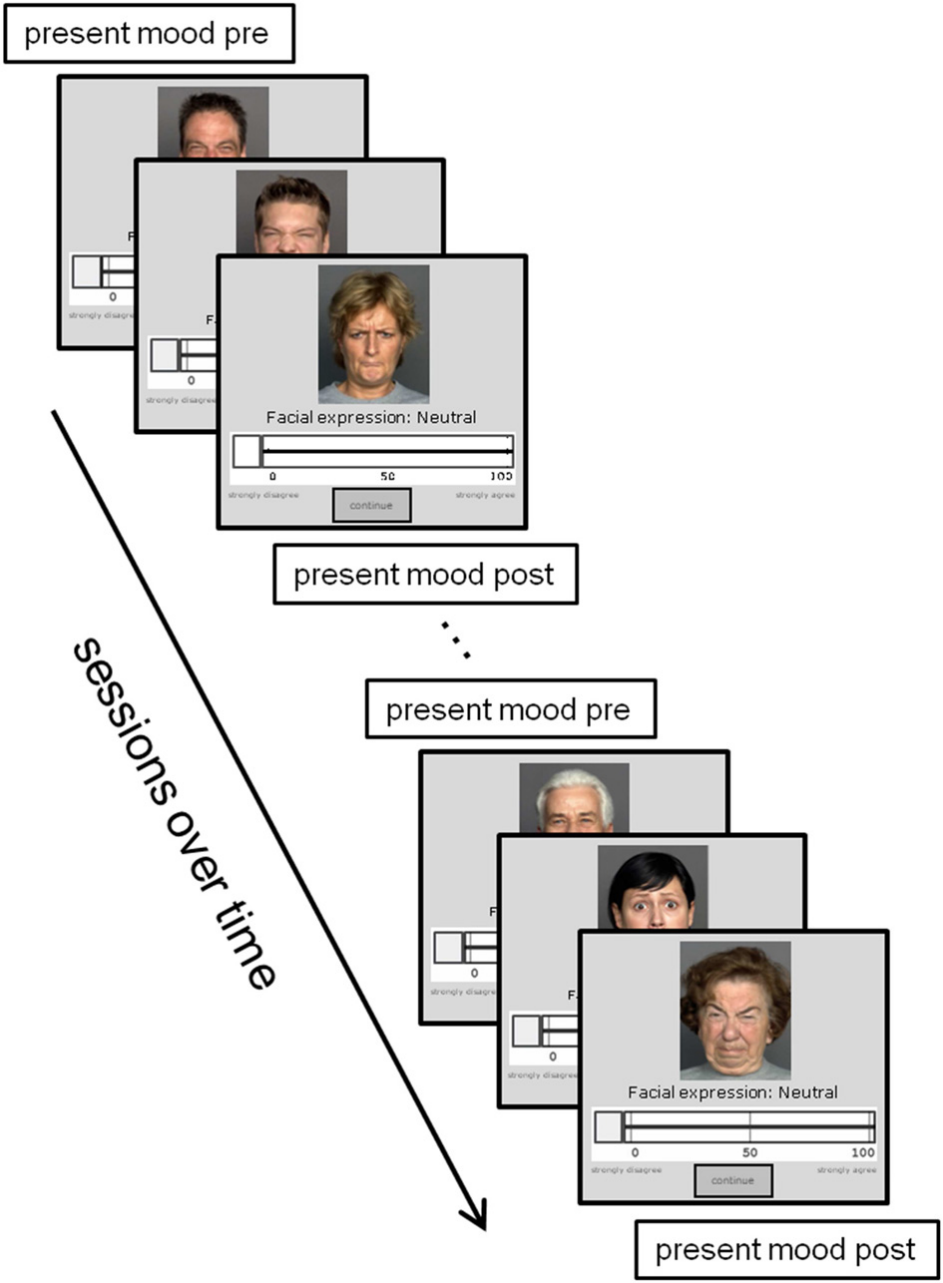
**Illustration of the study procedure**. Present mood was assessed at the beginning and the end of each session. During each session, participants rated the emotional expression of different face pictures by adjusting a slider. In addition, the primary emotional expression (happiness, sadness, anger, disgust, fear, or neutrality) was assessed via categorical rating.

The study was approved by the MPI ethics review board. Figure 1 provides a graphical illustration of the study procedure. After giving informed consent, participants were randomly assigned to one of two sets of parallel face pictures, which were presented one at a time on a 19-inch monitor. For each picture, participants indicated in a self-paced fashion the degree of happiness, sadness, fear, disgust, anger, or neutrality perceived in the face on a scale from 0 (*does not apply at all*) to 100 (*applies completely*). After these continuous ratings, participants indicated which of the six emotions was primarily displayed in the face (categorical rating). This was followed by additional face-specific questions that are not of relevance for the present paper. Due to the large number of face stimuli, participants had to complete the ratings in several sessions. Each session lasted for 100 min and there was only one session per day. Although it took up to 24 sessions for the slowest participant to complete all ratings, we restricted our analyses to the first 10 sessions to maintain comparability with previous research and to avoid increasing sparseness of the data. Participants' mood was assessed at the beginning and at the end of each session.

### Measures

Positive and negative mood was assessed using Hampel's (1977) *Adjective Scales to Assess Mood*. For positive mood, participants indicated on a scale from one to five the extent to which they currently felt happy, cheerful, elated, in high spirits, relaxed, mellow, and exuberant. Negative mood was assessed using the seven items insecure, sorrowful, disappointed, hopeless, melancholic, downhearted, and helpless. Corrected item-scale correlations for positive mood at the first measurement time point ranged between 0.51 and 0.79 (Cronbach's α = 0.89) and between 0.41 and 0.63 (Cronbach's α = 0.81) for negative mood (see Voelkle et al., 2013).

### Stimuli

Face photographs of 58 younger (19–31 years), 56 middle-aged (39–55 years), and 57 older (69–80 years) adults were used as stimulus material. The photographs were taken from the FACES Lifespan Database of Facial Expressions (Ebner et al., 2010), which contains in two parallel sets a total of 2052 pictures, displaying each of the 171 target persons with a prototypical happy, angry, sad, disgusted, fearful, or neutral facial expression (i.e., the pictures were taken at the peak of the emotional expression, following a standardized production and selection procedure in line with the Affect Program Theory of facial expressions by Ekman, 1993). In line with the race and ethnicity of study participants and in order to reduce the design complexity (e.g., by avoiding possible race-biased in-group vs. out-group effects; cf. Meissner and Brigham, 2001) all face models were Caucasian. For details on the construction of the FACES database see Ebner et al. (2010).

### Analysis

For each of the 1026 photographs (171 individuals times six emotions), *N* = 154 participants were asked to provide continuous emotion ratings on six different emotions, resulting in a theoretical maximum of 1026 × 6 × 154 = 948,024 ratings (plus the categorical emotion ratings and other ratings as mentioned above). Given that the face pictures displayed maximally prototypical emotional expressions, with about 80%^3^, the average recognition rate of the primary emotional expression was fairly high. As noted before, we were not interested in emotion recognition, but in the likelihood of ascribing an additional emotional expression to a face, for which the primary emotion had already been correctly recognized. Therefore, we controlled for interindividual differences in accuracy of emotion recognition, by including only ratings of those stimuli of which the primary emotional expression had been correctly identified (as indicated by the categorical ratings). Furthermore, we decided to analyze the *likelihood* with which an additional emotional expression was perceived in a face rather than the *intensity* of this perception. The reasons for this decision were twofold: First, the ratings of the additional emotions attributed to a face followed a highly right-skewed distribution. Despite correctly recognizing the primary facial expressions, some individuals indicated additional emotions of 100 (highest intensity of emotion expression) on a scale from 0 to 100. In addition, some people seemed to have used the middle of the rating scale as a reference point for some of their ratings. As a consequence, we could not find a meaningful transformation (such as a log-transform) that would have resulted in homoscedastic and normally distributed residuals. Second, in about 74% of the ratings, no additional continuous emotion ratings were provided resulting in a preponderance of zeros. Although models have been developed to deal with the combination of a zero-inflated and continuous or count part (Hall, 2000; Olsen and Schafer, 2001), we are not aware of any readily available integration with crossed random effects analyses employed in the present paper. Future research in this direction will be desirable.

Differences in the likelihood to perceive an emotion in addition to the primary emotion were modeled as a function of the type of *emotion* (happiness, sadness, fear, disgust, anger, neutrality; dummy coded with neutrality as baseline), *mood* at the beginning of the session (group mean centered at the average mood level prior to each session), study *session* (1–10; grand mean centered at 5.5), *age group* of perceiver (younger, middle-aged, older; effects coded), *stimulus number* (i.e., the relative position of a face photograph in the sequence of photographs presented to an individual participant, centered at the average number of stimuli rated by each individual in each session), and interactions between these factors^4^. Faces and participants were treated as two freely estimated crossed random effects in a generalized linear mixed effects model with a logit link function using the lme4 package 1.0.4 (Bates et al., 2013; see also Pinheiro and Bates, 2000) of R version 2.15.2 (R Core Team, 2012).

The possible role of emotion perception in faces as a mood-regulatory function was investigated by predicting the pre- to post-session *changes* in mood by how often a participant indicated perceiving a certain emotional expression out of the total number of ratings of this participant (i.e., the individual percentage of emotion perception in each session). In previous work we have demonstrated that changes in pre- to post-session mood decline significantly across the course of the entire study and that this change-in-change is almost perfectly captured by a logistic growth curve model (Voelkle et al., 2013). In order to control for this general trend we used the same model as in Voelkle et al. (2013) and added the interaction between age group and percentage of perceived emotional expression as time varying covariates with time varying effects^5^. To control for overall trends in emotion perception, session-mean centered scores were used.

## Results

The results section is organized as follows: Table 1 contains all parameter estimates of a single crossed random effects model as described above. In the following, we report results on the relationship between *positive* mood and emotion perception in faces. The corresponding results regarding *negative* mood and emotion perception are presented in Table 2. We will begin with discussing differences between displayed emotions, changes in emotion perception across study sessions, and age group differences in the perception of different emotions (Tables 1, 2, Part A). Next, we will turn to the question of *mood-congruent vs. mood-incongruent information processing in faces* by investigating the effect of present mood on the perception of different emotions in faces (*Hypothesis 1*; Tables 1, 2, Part B), as well as *age group differences in mood-congruent vs. mood-incongruent information processing* (*Hypothesis 2*; Tables 1, 2, Part C). After that we will focus on *the role of the temporal sequence in emotion processing* by analyzing the effect of stimulus position on emotion perception across the six different facial expressions along with a short discussion of possible three-way interactions of mood × stimulus position × emotion (*Hypothesis 3*; Tables 1, 2, Part D). Finally, we will investigate the *effectiveness of emotion perception in faces as a mood-regulatory function* (*Hypothesis* 4; **Figure 4**).

**Table 1:** **Results of a crossed random effects analysis predicting the likelihood of perceiving an (additional but the primary) emotional expression in a face by type of emotion, positive mood, session number, stimulus number, and age group**.

**Parameter**	**Estimate**	***SE***	***t*-value**	***p*-value**
**(A)**				
Intercept (baseline: neutrality)	−1.9777	0.1230	−16.07	0.000
Happiness	−0.7191	0.018	−39.94	0.000
Anger	1.0122	0.0147	69.03	0.000
Disgust	0.7639	0.0147	51.88	0.000
Sadness	0.7937	0.0147	53.90	0.000
Fear	0.9049	0.0147	61.53	0.000
Session	−0.1340	0.0016	−83.71	0.000
Session squared	0.0173	0.0006	29.25	0.000
Younger adults (baseline: neutrality)	−0.3635	0.1727	−2.10	0.035
Older adults (baseline: neutrality)	0.1622	0.1736	0.93	0.350
Happiness × younger adults	−0.0446	0.025	−1.78	0.075
Happiness × older adults	−0.0179	0.0265	−0.68	0.499
Anger × younger adults	0.0717	0.0202	3.55	0.000
Anger × older adults	0.0619	0.0216	2.87	0.004
Disgust × younger adults	0.1361	0.0203	6.72	0.000
Disgust × older adults	0.0318	0.0217	1.47	0.142
Sadness × younger adults	0.1819	0.0203	8.98	0.000
Sadness × older adults	−0.0182	0.0217	−0.84	0.403
Fear × younger adults	0.0727	0.0203	3.59	0.000
Fear × older adults	0.1388	0.0216	6.41	0.000
**(B) MOOD−CONGRUENT vs. MOOD−INCONGRUENT INFORMATION PROCESSING IN FACES**				
Positive mood (baseline: neutrality)	0.0481	0.0155	3.11	0.002
Positive mood × happiness	0.1177	0.0207	5.69	0.000
Positive mood × anger	−0.0365	0.0167	−2.19	0.029
Positive mood × disgust	−0.0595	0.0167	−3.56	0.000
Positive mood × sadness	−0.0560	0.0167	−3.34	0.001
Positive mood × fear	−0.1086	0.0167	−6.50	0.000
**(C) AGE GROUP DIFFERENCES IN MOOD−CONGRUENT vs. MOOD−INCONGRUENT INFORMATION PROCESSING**				
Positive mood × younger adults (baseline: neutrality)	0.0305	0.0204	1.50	0.135
Positive mood × older adults (baseline: neutrality)	−0.1302	0.0236	−5.53	0.000
Positive mood × happiness × younger adults	−0.1567	0.0288	−5.44	0.000
Positive mood × happiness × older adults	0.1512	0.0305	4.96	0.000
Positive mood × anger × younger adults	−0.0128	0.0227	−0.56	0.573
Positive mood × anger × older adults	0.0056	0.0244	0.23	0.818
Positive mood × disgust × younger adults	−0.0580	0.0228	−2.55	0.011
Positive mood × disgust × older adults	0.0764	0.0245	3.12	0.002
Positive mood × sadness × younger adults	0.0344	0.0228	1.51	0.131
Positive mood × sadness × older adults	0.0810	0.0246	3.29	0.001
Positive mood × fear × younger adults	0.0149	0.0228	0.65	0.513
Positive mood × fear × older adults	−0.0291	0.0244	−1.19	0.234
**(D) TEMPORAL SEQUENCE IN MOOD−CONGRUENT vs. MOOD−INCONGRUENT INFORMATION PROCESSING**				
Stimulus number (baseline: neutrality)	−0.0004	0.0004	−1.05	0.295
Happiness × stimulus number	−0.0028	0.0006	−4.43	0.000
Anger × stimulus number	−0.0026	0.0005	−4.97	0.000
Disgust × stimulus number	−0.0029	0.0005	−5.63	0.000
Sadness × stimulus number	−0.0016	0.0005	−3.09	0.002
Fear × stimulus number	−0.0017	0.0005	−3.36	0.001
Positive mood × stimulus number (baseline: neutrality)	−0.0008	0.0005	−1.75	0.079
Positive mood × happiness × stimulus number	0.0011	0.0007	1.49	0.137
Positive mood × anger × stimulus number	0.0014	0.0006	2.36	0.018
Positive mood × disgust × stimulus number	0.0014	0.0006	2.44	0.015
Positive mood × sadness × stimulus number	0.0006	0.0006	1.01	0.314
Positive mood × fear × stimulus number	0.0010	0.0006	1.63	0.102
Random intercept face	Variance = 0.016; *SD* = 0.127			
Random intercept participant	Variance = 2.289; *SD* = 1.513			
AIC	379620.8			
BIC	380192.4			
Log-likelihood	−189758.4			
Deviance	379516.8			

*Type of emotion was dummy coded with perception of neutral emotional expression as baseline. Session = grand mean centered session number; Session squared = squared grand mean centered session number; Age group of participant (younger, middle-aged, older adults) was effect coded; Positive mood was group mean centered by subtracting the average positive mood at each study session; Stimulus number = individually centered position of stimulus in the sequence of stimuli within each session. Faces and participants were treated as two crossed random effects*.

**Table 2:** **Results of a crossed random effects analysis predicting the likelihood of perceiving an (additional but the primary) emotional expression in a face by type of emotion, negative mood, session number, stimulus number, and age group**.

**Parameter**	**Estimate**	***SE***	***t*-value**	***p*-value**
**(A)**				
Intercept (baseline: neutrality)	−1.9633	0.1228	−15.98	0.000
Happiness	−0.7409	0.0190	−39.06	0.000
Anger	0.9784	0.0151	64.99	0.000
Disgust	0.7296	0.0151	48.26	0.000
Sadness	0.7607	0.0151	50.32	0.000
Fear	0.8637	0.0151	57.13	0.000
Session	−0.1331	0.0016	−82.89	0.000
Session squared	0.0174	0.0006	29.29	0.000
Younger adults (baseline: neutrality)	−0.3565	0.1724	−2.07	0.039
Older adults (baseline: neutrality)	0.1484	0.1734	0.86	0.392
Happiness × younger adults	−0.0519	0.0266	−1.95	0.051
Happiness × older adults	−0.0735	0.0286	−2.56	0.010
Anger × younger adults	0.0529	0.021	2.52	0.012
Anger × older adults	0.0877	0.0221	3.96	0.000
Disgust × younger adults	0.0956	0.0211	4.53	0.000
Disgust × older adults	0.0631	0.0222	2.84	0.005
Sadness × younger adults	0.1713	0.0211	8.13	0.000
Sadness × older adults	0.0018	0.0223	0.08	0.936
Fear × younger adults	0.0169	0.0211	0.80	0.423
Fear × older adults	0.1773	0.0222	7.97	0.000
**(B) MOOD−CONGRUENT vs. MOOD−INCONGRUENT INFORMATION PROCESSING IN FACES**				
Negative mood (baseline: neutrality)	0.0132	0.0265	0.50	0.618
Negative mood × happiness	−0.2103	0.0396	−5.31	0.000
Negative mood × anger	−0.0520	0.0299	−1.74	0.081
Negative mood × disgust	0.0514	0.0298	1.72	0.085
Negative mood × sadness	−0.0500	0.0299	−1.67	0.094
Negative mood × fear	0.1490	0.0297	5.03	0.000
**(C) AGE GROUP DIFFERENCES IN MOOD−CONGRUENT vs. MOOD−INCONGRUENT INFORMATION PROCESSING**				
Negative mood × younger adults (baseline: neutrality)	−0.0585	0.0314	−1.86	0.063
Negative mood × older adults (baseline: neutrality)	0.0515	0.0422	1.22	0.222
Negative mood × happiness × younger adults	0.3286	0.0463	7.09	0.000
Negative mood × happiness × older adults	−0.5495	0.0654	−8.40	0.000
Negative mood × anger × younger adults	0.2393	0.0357	6.70	0.000
Negative mood × anger × older adults	0.0460	0.0459	1.00	0.316
Negative mood × disgust × younger adults	0.2511	0.0357	7.04	0.000
Negative mood × disgust × older adults	−0.0853	0.046	−1.86	0.063
Negative mood × sadness × younger adults	0.1920	0.0358	5.37	0.000
Negative mood × sadness × older adults	−0.0669	0.0461	−1.45	0.147
Negative mood × fear × younger adults	0.2346	0.0355	6.60	0.000
Negative mood × fear × older adults	−0.0040	0.0458	−0.09	0.931
**(D) TEMPORAL SEQUENCE IN MOOD−CONGRUENT vs. MOOD−INCONGRUENT INFORMATION PROCESSING**				
Stimulus number (baseline: neutrality)	−0.0005	0.0004	−1.38	0.167
Happiness × stimulus number	−0.0026	0.0006	−4.31	0.000
Anger × stimulus number	−0.0024	0.0005	−4.70	0.000
Disgust × stimulus number	−0.0028	0.0005	−5.51	0.000
Sadness × stimulus number	−0.0015	0.0005	−3.03	0.002
Fear × stimulus number	−0.0016	0.0005	−3.13	0.002
Negative mood × stimulus number (baseline: neutrality)	−0.0002	0.0007	−0.36	0.720
Negative mood × happiness × stimulus number	0.0011	0.0010	1.06	0.289
Negative mood × anger × stimulus number	0.0005	0.0008	0.64	0.525
Negative mood × disgust × stimulus number	0.0013	0.0008	1.53	0.126
Negative mood × sadness × stimulus number	0.0009	0.0008	1.04	0.297
Negative mood × fear × stimulus number	0.0002	0.0008	0.29	0.769
Random intercept face	Variance = 0.016; *SD* = 0.127			
Random intercept participant	Variance = 2.280; *SD* = 1.510			
AIC	379195.9			
BIC	379767.5			
Log-likelihood	−189546.0			
Deviance	379091.9			

*Type of emotion was dummy coded with perception of neutral emotional expression as baseline. Session = grand mean centered session number; Session squared = squared grand mean centered session number; Age group of participant (younger, middle-aged, older adults) was effect coded; Negative mood was group mean centered by subtracting the average negative mood at each study session; Stimulus number = individually centered position of the stimulus in the sequence of stimuli within each session. Faces and participants were treated as two crossed random effects*.

After recognizing the primary emotional expression, the average probability to perceive an additional neutral expression in a randomly selected face picture, presented after half of the stimuli had been rated within a given study session, and after half of the sessions had been completed was [100 · (1/(1 + e^1.978^))] = 12.16% in a participant with an average within-session positive mood. However, there were large differences between the types of emotions: While the probability of perceiving an additional happy expression was significantly lower [100 · (1/(1 + e^1.978 + 0.719^))] = 6.31% (logit = −0.719; odds ratio = 0.487; *p* < 0.001), the probability of perceiving an additional angry expression was significantly higher (27.58%; logit = 1.012; odds ratio = 2.751; *p* < 0.001). The other emotions fell somewhere in between. Likewise, the likelihood of reporting an additional (neutral) emotional expression changed as a function of the number of sessions, with a significantly higher likelihood at the beginning of the study (20.82%) than after 10 sessions (6.79%). Note that the decline was not linear but leveled off toward the end of the study (i.e., a positive quadratic effect; logit_Linear_ = −0.1340, odds ratio = 0.875, *p* < 0.001; logit_Quadratic_ = 0.017, odds ratio = 1.017, *p* < 0.001). In contrast to the impact of session and type of emotion, age group differences in the perception of different emotions were smaller and somewhat mixed. As compared to the average age (age groups are effects coded^6^), the likelihood to perceive anger increased for both, younger (logit_Younger_ = 0.072, odds ratio = 1.07, *p* < 0.001) and older (logit_Older_ = 0.062, odds ratio = 1.06, *p* < 0.001) adults. The same applied to fear (logit_Younger_ = 0.073, odds ratio = 1.08, *p* < 0.001; logit_Older_ = 0.139, odds ratio = 1.15, *p* < 0.001). In addition, the likelihood of reporting disgust and sadness increased significantly in younger adults. However, with an odds ratio of e^0.182^ = 1.20 even the largest effect was rather small. See Table 1, Part A, for details.

### Mood-congruent vs. mood-incongruent information processing in faces

Turning to *Hypothesis 1*, and putting age aside for the moment, there was clear evidence for a mood-congruency effect in emotion perception. The more positive participants' mood, the more likely they were to perceive happiness in the presented faces. For example, someone in a maximally positive mood would have a probability of [100 · (1/(1 + e^1.978 + 0.719 − 0.048 · 2 − 0.118 · 2^))] = 8.58% of perceiving an additional happy expression, but only a probability of [100 · (1/(1 + e^1.978 + 0.719 − 0.048 · −2 − 0.118 · −2^))] = 4.61% if scoring at the lower end of the mood scale (logit = 0.118; odds ratio = 1.125; *p* < 0.001). Being in a positive mood also slightly increased the probability of perceiving an additional neutral expression (logit = 0.048; odds ratio = 1.049; *p* < 0.01). However, being in a positive mood consistently reduced the probability of perceiving an additional expression for all negatively valenced emotions (i.e., anger, disgust, sadness, and fear; see Table 1). See Table 1, Part B.

As apparent from Table 2, a similar pattern held for increasing negative mood. That is, higher levels of negative mood led to a lower probability of perceiving an additional happy expression (logit = −0.210; odds ratio = 0.810; *p* < 0.001), but an increase in the likelihood to perceive an additional fearful expression (logit = 0.149; odds ratio = 1.161; *p* < 0.001). All other emotions fell somewhere in between (none of them were significant at a 0.05 alpha level).

### Age group differences in mood-congruent vs. mood-incongruent information processing

**Figure 2:**
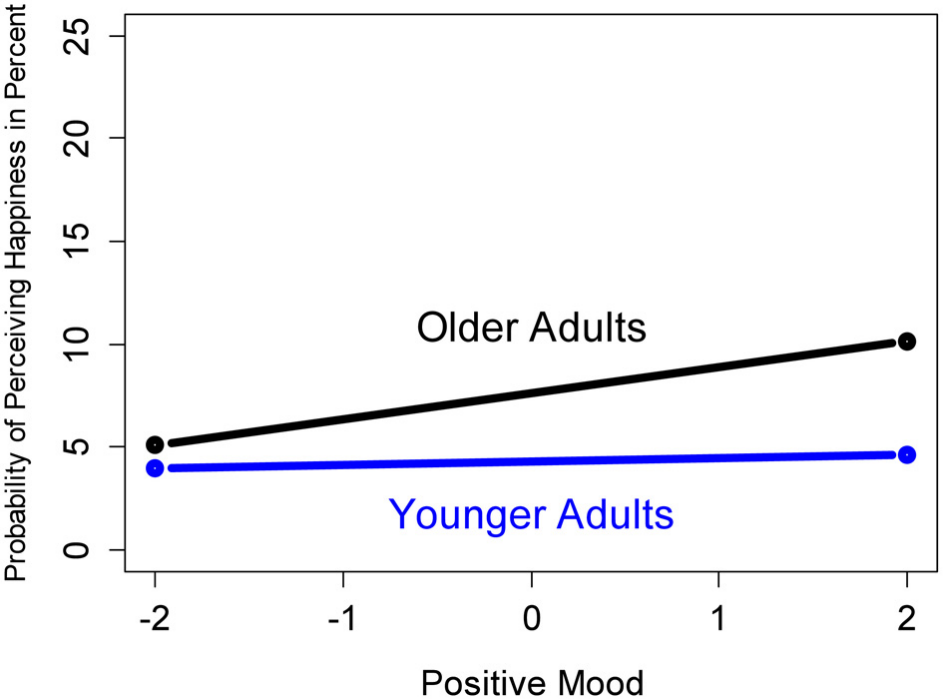
**Predicted probability of perceiving an additional happy emotional expression in faces for younger and older adults with maximally high (right) vs. maximally low (left) levels of positive mood**.

**Figure 3:**
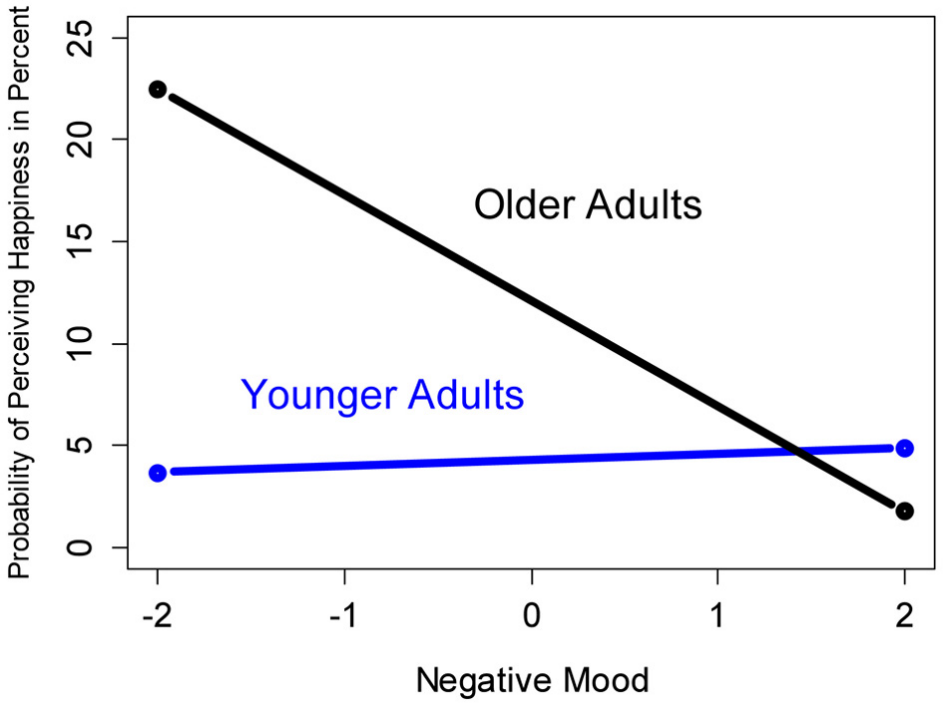
**Predicted probability of perceiving an additional happy emotional expression in faces for younger and older adults with maximally high (right) vs. maximally low (left) levels of negative mood**.

Contradictory to *Hypothesis 2*, which predicted that older adults have a higher likelihood of perceiving positive emotions in facial expressions when being in a bad mood (i.e., mood-*in*congruency), a mood-congruency effect was primarily shown by older adults. Other than younger adults (logit = −0.157; odds ratio = 0.855; *p* < 0.001), older adults exhibited a significantly higher probability of perceiving happiness, when being in a good mood (logit = 0.151; odds ratio = 1.163; *p* < 0.001). Figure 2 provides a graphical illustration of the model-predicted probabilities of perceiving an additional happy emotional expression in faces for younger and older adults with maximally high vs. low levels of positive mood. As compared to the baseline, higher levels of positive mood in older adults also increased the likelihood of perceiving disgust and sadness (logit = 0.076; odds ratio = 1.079; *p* < 0.01; logit = 0.081; odds ratio = 1.084; *p* < 0.01, respectively). In contrast, positive mood in older adults decreased the likelihood of perceiving neutrality (logit = −0.130; odds ratio = 0.878; *p* < 0.001). Although the results were somewhat mixed, the pronounced mood-congruency effect for older adults in the perception of happiness speaks against *Hypothesis 2*. See Table 1, Part C, for details. The effect for negative mood (Table 2) further bolstered the rejection of *Hypothesis 2*: Higher levels of negative mood in older adults *decreased* the probability of perceiving happiness (logit = −0.550; odds ratio = 0.578; *p* < 0.001), while no such decrease was found for younger adults (logit = 0.329; odds ratio = 1.390; *p* < 0.001). These results are illustrated in Figure 3.

### The role of the temporal sequence in emotion processing

The probability of perceiving an additional emotional expression decreased not only across study sessions, but also within each study session (i.e., with stimulus number; see Table 1, Part D). While the decrease was somewhat lower and non-significant for the perception of neutrality (logit = −0.0004; odds ratio = 0.999; *p* = 0.295), it was significant for all other emotions (*p* < 0.01). At the descriptive level, the downward trend in the likelihood of reporting an emotional expression, other than neutrality, was slightly weakened for participants in a positive mood, but none of the effects was significant at a 0.01 alpha level. Likewise, there were no three-way interactions with negative mood (see Table 2). Given the non-significant interaction of positive mood with stimulus number and negative mood with stimulus number, respectively, along with the uniform downward trend in the likelihood of perceiving an additional emotional expression, there was little evidence for a shift from mood-congruent to mood-incongruent information processing as a function of elapsed processing time in the present study. Thus, *Hypothesis 3* was rejected.

### Effectiveness of emotion perception in faces as a mood-regulatory function

**Figure 4:**
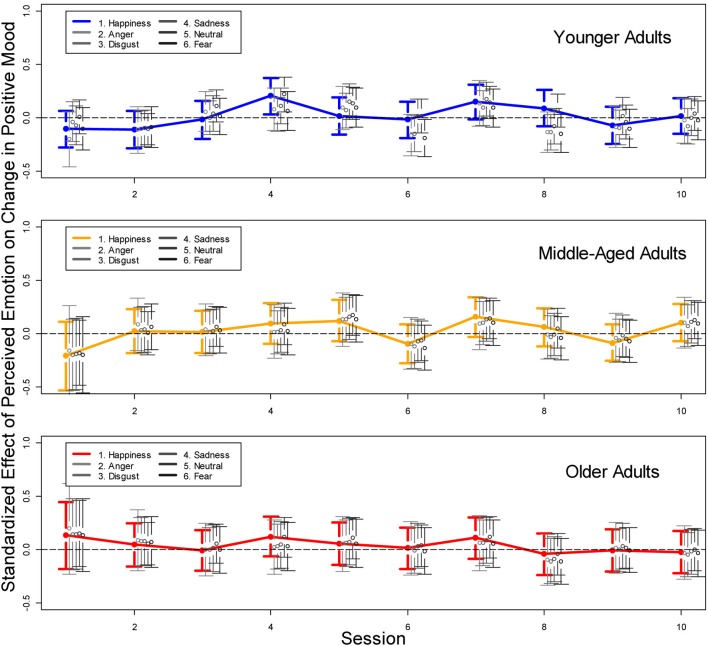
**Standardized effects of perceived happiness, anger, disgust, sadness, neutrality, and fear on changes between pre- to post-session mood for younger, middle-aged, and older participants**. In six separate analyses (for the six emotions), the interaction between perceived emotional expression and age group was entered as a time-varying covariate with time-varying effects into a logistic growth curve model of changes in mood changes across the 10 study sessions (see Voelkle et al., 2013).

In *Hypothesis* 4 we postulated a positive relationship between the likelihood of perceiving positively valenced emotions in faces and subsequent improvements in mood. This hypothesis was based on prior research suggesting that mood-incongruent information processing in older adults, or a shift from mood-congruent to mood-incongruent information processing as a function of processing time, may serve a mood-regulatory function—in particular if initial mood was bad. Given that we found no empirical support for Hypotheses 2 and 3 this seemed unlikely. To explicitly test the effect of emotion perception during a study session on mood changes from the beginning to the end of a session, we added the interaction between age group and percentage of perceived emotional expressions as time varying covariates to a logistic growth curve model of mood changes as described above. The results are presented in Figure 4 which shows the standardized effects of emotion perception on pre- to post-session mood changes across the 10 study sessions, separated for younger, middle-aged, and older adults, and for all six emotional expressions. As apparent from the 95% confidence intervals, almost none of the effects were significant and there was no consistent pattern across time, across emotions, or across age groups. Rather, most effects varied around zero, providing no empirical support for *Hypothesis 4*.

## Discussion

We began this work by asking how the way we feel influences the perception of the world around us, and how this perception may affect our own feelings. We approached this question by studying the relationship between natural mood (as opposed to experimentally induced mood) and the attribution of emotions to faces for which the primary emotional expression had been correctly identified.

Consistent with our expectations in Hypothesis 1, more positive mood increased the likelihood of perceiving a happy facial expression. In particular older adults had a higher likelihood of reporting the perception of happiness in faces when being in a positive mood, and a lower likelihood when being in a negative mood. Likewise, older adults were increasingly less likely to perceive happiness in faces when in a more negative mood. Both findings did not apply to younger adults. This stands in contrast to Hypothesis 2 which predicted that older adults have a higher likelihood of perceiving positive emotions in facial expressions when in a bad mood. In contrast to previous reports in the literature (Forgas and Ciarrochi, 2002; Isaacowitz et al., 2008, 2009a), we found neither evidence for a change from mood-congruent to mood-incongruent responses over time (Hypothesis 3) nor for a mood-regulatory effect of emotion processing in faces (Hypothesis 4). In the remainder of the discussion we will offer interpretations for our central findings.

### Mood-congruent information processing

Although partly in contrast to our expectations based on previous research using active mood induction in controlled laboratory settings, our results may be explained in terms of the AIM. The fact that older adults showed a mood-congruency effect in emotion perception suggests their use of a high affect infusion strategy such as heuristic or substantive processing. Unfortunately, the present study does not allow us to clearly disentangle the underlying mechanisms in terms of heuristic vs. substantive processing. Even though this remains an important topic to be addressed in future research, we believe that the observed mood-congruent information processing in older adults may even be due to a combination of the two processing strategies. On the one hand—and independent of present mood—older compared to younger adults were not only more likely to indicate the perception of an additional happy but also an additional neutral facial expression (see Tables 1, 2). This may reflect their higher personal involvement in the task, which suggests the use of a substantive processing strategy. On the other hand, reducing cognitive resources may have let older adults rely stronger on other sources of information, such as their current mood, supporting the notion of a heuristic (mood-as-information) processing strategy.

For younger adults in contrast, the increase in the perception of happiness almost completely disappeared (see Figure 2), suggesting their use of a low affect infusion strategy such as motivated processing or the direct access strategy. The motivation to increase one's mood—in particular when in a bad mood—should not only eliminate a mood-congruency effect, but should result in mood-incongruent information processing (i.e., an increased likelihood of perceiving happiness). The fact that we did not observe such an effect, rather suggests the use of a direct access strategy in younger adults. As described in the introduction, this strategy is particularly likely when “the judge is not personally involved, and there are no strong cognitive, affective, motivational, or situational forces mandating more elaborate processing” (Forgas, 1995, p. 46).

### Mood-incongruent information processing as a mood-regulatory function

As apparent from Figure 4, there was no empirical support that emotion perception in faces serves a mood-regulatory function under conditions of natural occurring mood rather than actively induced mood. This stands in contrast to research using experimentally induced mood, which showed that the focus of older adults in a bad mood on positively valenced faces may help to regulate their mood. One reason for these different findings may be that the naturally occurring fluctuations in positive mood assessed in the present context were not sufficiently strong to reveal such an effect. Furthermore, ascribing an additional emotional expression to a face whose primary emotion had been correctly recognized is, of course, a much more subtle measure of mood-(in)congruent emotion processing as compared, for example, to gaze patterns toward prototypically positively or negatively valenced facial expressions as used in the study by Isaacowitz et al. (2008). In addition, the anticipatory (down)adjustment of positive mood may have further reduced the likelihood of discovering such an effect (Voelkle et al., 2013).

### Limitations and future directions

We believe the general downward trend in the likelihood of reporting an emotional expression is likely due to participant's increasing fatigue and decreasing motivation. However, it is a shortcoming of the present study that these factors were not assessed in self-report, thus this belief remains speculative. Although we statistically controlled for the downward trend, the remaining variability in mood and emotion perception may have been too small to allow for additional mood regulation effects by means of emotion perception. After all, if an individual's mood is already perfectly adjusted to the situation at hand—or if fatigue effects are very strong—there is little room for additional regulation via mood-incongruent information processing.

This may be viewed as a shortcoming of the present study. However, it also shows that while natural mood (as opposed to experimentally induced mood) is likely to affect the perception of emotions in faces, a possible mood-regulatory effect of such perception seems negligible. In fact, we found that in particular for older adults, positive mood may rather increase the likelihood of perceiving positively valenced facial expressions.

The somewhat undifferentiated take on emotions and mood may be considered another weakness of the study. Future research may profit from more fine-grained distinctions between different aspects of positive and negative mood, including different aspects of valence and arousal, and a discrete emotion perspective as outlined by Kunzmann et al. (2014) in this issue. However, the primary purpose of the present paper was not to offer a comprehensive framework on age-related changes in the intricate interplay between specific aspects of affective experience and the perception (attribution) of additional emotions in faces whose primary emotion were correctly specified. Rather the focus was to place research on emotion perception in faces into the broader context on mood-congruent vs. mood-incongruent information processing. To accomplish this goal it is necessary to remain at a more general level, although we do report more detailed findings. In the same way we encourage future research to focus on more detailed aspects of experienced mood and the perception of emotional expression, we, thus, also encourage more integrative research linking these findings back to more general research on age-related changes in mood-(in)congruent information processing. We hope the present paper will contribute to both ends.

### Conflict of interest statement

The authors declare that the research was conducted in the absence of any commercial or financial relationships that could be construed as a potential conflict of interest.

## Footnotes

^1^For an analysis of age differences in emotional expression identification as a function of perceiver's and face model's age and facial expression with the present data, we refer the reader to Ebner et al. (2010).

^2^Although we disentangle the perception of additional emotional expressions from emotion recognition by analyzing only correctly recognized emotions, this does not preclude the possibility that people with higher recognition ability (and possible age differences therein) may be less likely to ascribe additional emotional expressions to a face after having identified the primary emotional expression. To control for this possibility, we repeated all analyses by including the person-specific recognition ability as an additional covariate. As a matter of fact, higher recognition ability decreased the likelihood of perceiving an additional emotional expression significantly (logit = −10.750; *p* < 0.001). In addition, the slightly lower (and non-significant at a 0.01 alpha level) likelihood of younger adults of perceiving an additional emotional expression disappeared after controlling for recognition ability. All other results, however, remained virtually unaffected by controlling for person-specific recognition ability. The additional analyses are provided as online Supplementary Materials A and B.

^3^The average recognition rate of emotional expressions differed across emotions and age groups. For a more detailed analysis, see Ebner et al. (2010).

^4^In a separate analysis, we included sex of participant as an additional predictor, which resulted in a non-significant improvement in model fit and did not change the pattern of results or any of the substantive conclusions (2Log-Likelihood_*Diff*_ = 1.2; df_*Diff*_ = 1). Because we had no prior expectations regarding sex effects, we report the analyses without sex.

^5^Missing values on the predictor variables due to study drop out were handled via Expectation Maximization (EM) imputation.

^6^Middle-aged adults were chosen as the base group. As pointed out by Cohen et al. (2003), when using effects coding the base group is usually selected to be the group “for which comparisons to the mean are of *least* interest” (p. 322). This is because the analysis does not inform us *directly* about this group, but only indirectly. All information, however, is considered in the analysis. See Cohen et al. (2003) for more detailed information.
