# Supplementary material for: A note on age differences in mood-congruent vs. mood-incongruent emotion processing in faces
Source: Front Psychol. 2014 Jun 26;5:635. doi: 10.3389/fpsyg.2014.00635 (PMC4071858; doi:10.3389/fpsyg.2014.00635)
Supplement: Supplementary file 1 [file DataSheet1.DOCX]

**Supplementary Material A**

*Results of a Crossed Random Effects Analysis Predicting the Likelihood of Perceiving an (Additional but the Primary) Emotional Expression in a Face by Type of Emotion, Positive Mood, Session Number, Stimulus Number, Age Group, Controlling for Recognition Ability.*

| Parameter | Estimate |  | Std. Error | *t*-value | *p*-value |
| --- | --- | --- | --- | --- | --- |
| *A)* | | | | |  |
| Intercept (Baseline: Neutrality) | -1.9790 |  | 0.1051 | -18.84 | 0.000 |
| Happiness | -0.7191 |  | 0.0180 | -39.94 | 0.000 |
| Anger | 1.0120 |  | 0.0147 | 69.03 | 0.000 |
| Disgust | 0.7639 |  | 0.0147 | 51.88 | 0.000 |
| Sadness | 0.7937 |  | 0.0147 | 53.9 | 0.000 |
| Fear | 0.9049 |  | 0.0147 | 61.53 | 0.000 |
| Session | -0.1340 |  | 0.0016 | -83.69 | 0.000 |
| Session Squared | 0.0173 |  | 0.0006 | 29.25 | 0.000 |
| Younger Adults (Baseline: Neutrality) | -0.0088 |  | 0.1544 | -0.06 | 0.955 |
| Older Adults (Baseline: Neutrality) | -0.2127 |  | 0.1560 | -1.36 | 0.173 |
| Happiness × Younger Adults | -0.0446 |  | 0.0250 | -1.78 | 0.075 |
| Happiness × Older Adults | -0.0180 |  | 0.0265 | -0.68 | 0.498 |
| Anger × Younger Adults | 0.0717 |  | 0.0202 | 3.55 | 0.000 |
| Anger × Older Adults | 0.0619 |  | 0.0216 | 2.87 | 0.004 |
| Disgust × Younger Adults | 0.1361 |  | 0.0203 | 6.72 | 0.000 |
| Disgust × Older Adults | 0.0318 |  | 0.0217 | 1.47 | 0.142 |
| Sadness × Younger Adults | 0.1819 |  | 0.0203 | 8.98 | 0.000 |
| Sadness × Older Adults | -0.0182 |  | 0.0217 | -0.84 | 0.403 |
| Fear × Younger Adults | 0.0727 |  | 0.0203 | 3.59 | 0.000 |
| Fear × Older Adults | 0.1388 |  | 0.0216 | 6.41 | 0.000 |
|  |  |  |  |  |  |
| *B) Mood-congruent versus mood-incongruent information processing in faces* | | | | |  |
| Positive Mood (Baseline: Neutrality) | 0.0477 |  | 0.0155 | 3.08 | 0.002 |
| Positive Mood × Happiness | 0.1177 |  | 0.0207 | 5.69 | 0.000 |
| Positive Mood × Anger | -0.0364 |  | 0.0167 | -2.19 | 0.029 |
| Positive Mood × Disgust | -0.0595 |  | 0.0167 | -3.56 | 0.000 |
| Positive Mood × Sadness | -0.0560 |  | 0.0168 | -3.34 | 0.001 |
| Positive Mood × Fear | -0.1085 |  | 0.0167 | -6.5 | 0.000 |
|  |  |  |  |  |  |
| *C) Age group differences in mood-congruent versus mood-incongruent information processing* | | | | |  |
| Positive Mood × Younger Adults (Baseline: Neutrality) | 0.0306 |  | 0.0204 | 1.5 | 0.133 |
| Positive Mood × Older Adults (Baseline: Neutrality) | -0.1286 |  | 0.0236 | -5.46 | 0.000 |
| Positive Mood × Happiness × Younger Adults | -0.1567 |  | 0.0288 | -5.44 | 0.000 |
| Positive Mood × Happiness × Older Adults | 0.1512 |  | 0.0305 | 4.97 | 0.000 |
|  |  |  |  |  |  |
| Parameter | Estimate |  | Std. Error | *t*-value | *p*-value |
| Positive Mood × Anger × Younger Adults | -0.0129 |  | 0.0227 | -0.57 | 0.569 |
| Positive Mood × Anger × Older Adults | 0.0056 |  | 0.0244 | 0.23 | 0.817 |
| Positive Mood × Disgust × Younger Adults | -0.0580 |  | 0.0228 | -2.55 | 0.011 |
| Positive Mood × Disgust × Older Adults | 0.0764 |  | 0.0245 | 3.12 | 0.002 |
| Positive Mood × Sadness × Younger Adults | 0.0343 |  | 0.0228 | 1.51 | 0.132 |
| Positive Mood × Sadness × Older Adults | 0.0810 |  | 0.0246 | 3.3 | 0.001 |
| Positive Mood × Fear × Younger Adults | 0.0148 |  | 0.0228 | 0.65 | 0.516 |
| Positive Mood × Fear × Older Adults | -0.0291 |  | 0.0244 | -1.19 | 0.234 |
|  |  |  |  |  |  |
| *D) Temporal sequence in mood-congruent versus mood-incongruent information processing* | | | | |  |
| Stimulus Number (Baseline: Neutrality) | -0.0004 |  | 0.0004 | -1.05 | 0.295 |
| Happiness × Stimulus Number | -0.0028 |  | 0.0006 | -4.43 | 0.000 |
| Anger × Stimulus Number | -0.0026 |  | 0.0005 | -4.97 | 0.000 |
| Disgust × Stimulus Number | -0.0029 |  | 0.0005 | -5.63 | 0.000 |
| Sadness × Stimulus Number | -0.0016 |  | 0.0005 | -3.09 | 0.002 |
| Fear × Stimulus Number | -0.0017 |  | 0.0005 | -3.36 | 0.001 |
|  |  |  |  |  |  |
| Positive Mood × Stimulus Number (Baseline: Neutrality) | -0.0008 |  | 0.0005 | -1.76 | 0.079 |
| Positive Mood × Happiness × Stimulus Number | 0.0011 |  | 0.0007 | 1.49 | 0.137 |
| Positive Mood × Anger × Stimulus Number | 0.0014 |  | 0.0006 | 2.36 | 0.018 |
| Positive Mood × Disgust × Stimulus Number | 0.0014 |  | 0.0006 | 2.44 | 0.015 |
| Positive Mood × Sadness × Stimulus Number | 0.0006 |  | 0.0006 | 1.01 | 0.314 |
| Positive Mood × Fear × Stimulus Number | 0.0010 |  | 0.0006 | 1.63 | 0.102 |
|  |  |  |  |  |  |
| Recognition Ability | -10.7500 |  | 1.4120 | -7.61 | 0.000 |
|  |  |  |  |  |  |
|  | | | | |  |
| Random Intercept Face | Variance = 0.016; Std.Dev. = 0.127 | | | | |
| Random Intercept Participant | Variance = 1.658; Std.Dev. = 1.287 | | | | |
| AIC | 379573.6 |  |  |  |  |
| BIC | 380156.2 |  |  |  |  |
| Log-Likelihood | -189733.8 |  |  |  |  |
| Deviance | 379467.6 |  |  |  |  |

*Note*. Type of emotion was dummy coded with perception of neutral emotional expression as baseline. Session = grand mean centered session number; Session squared = squared grand mean centered session number; Age group of participant (younger, middle-aged, older adults) was effect coded; Positive mood was group mean centered by subtracting the average positive mood at each study session; Stimulus number = individually centered position of stimulus in the sequence of stimuli within each session; Recognition ability = grand mean centered recognition ability per person. Faces and participants were treated as two crossed random effects.

**Supplementary Material B**

*Results of a Crossed Random Effects Analysis Predicting the Likelihood of Perceiving an (Additional but the Primary) Emotional Expression in a Face by Type of Emotion, Negative Mood, Session Number, Stimulus Number, Age Group, Controlling for Recognition Ability.*

| Parameter | Estimate |  | Std. Error | *t*-value | *p*-value |
| --- | --- | --- | --- | --- | --- |
| *A)* | | | | |  |
| Intercept (Baseline: Neutrality) | -1.9650 |  | 0.1041 | -18.88 | 0.000 |
| Happiness | -0.7410 |  | 0.0190 | -39.06 | 0.000 |
| Anger | 0.9784 |  | 0.0151 | 64.99 | 0.000 |
| Disgust | 0.7296 |  | 0.0151 | 48.26 | 0.000 |
| Sadness | 0.7607 |  | 0.0151 | 50.32 | 0.000 |
| Fear | 0.8637 |  | 0.0151 | 57.13 | 0.000 |
| Session | -0.1331 |  | 0.0016 | -82.89 | 0.000 |
| Session Squared | 0.0174 |  | 0.0006 | 29.29 | 0.000 |
| Younger Adults (Baseline: Neutrality) | 0.0048 |  | 0.1529 | 0.03 | 0.975 |
| Older Adults (Baseline: Neutrality) | -0.2332 |  | 0.1545 | -1.51 | 0.131 |
| Happiness × Younger Adults | -0.0519 |  | 0.0266 | -1.95 | 0.051 |
| Happiness × Older Adults | -0.0735 |  | 0.0287 | -2.57 | 0.010 |
| Anger × Younger Adults | 0.0528 |  | 0.0210 | 2.52 | 0.012 |
| Anger × Older Adults | 0.0878 |  | 0.0221 | 3.96 | 0.000 |
| Disgust × Younger Adults | 0.0956 |  | 0.0211 | 4.53 | 0.000 |
| Disgust × Older Adults | 0.0631 |  | 0.0222 | 2.84 | 0.005 |
| Sadness × Younger Adults | 0.1713 |  | 0.0211 | 8.13 | 0.000 |
| Sadness × Older Adults | 0.0018 |  | 0.0223 | 0.08 | 0.935 |
| Fear × Younger Adults | 0.0169 |  | 0.0211 | 0.8 | 0.425 |
| Fear × Older Adults | 0.1773 |  | 0.0222 | 7.98 | 0.000 |
|  |  |  |  |  |  |
| *B) Mood-congruent versus mood-incongruent information processing in faces* | | | | |  |
| Negative Mood (Baseline: Neutrality) | 0.0140 |  | 0.0265 | 0.53 | 0.598 |
| Negative Mood × Happiness | -0.2103 |  | 0.0396 | -5.31 | 0.000 |
| Negative Mood × Anger | -0.0519 |  | 0.0299 | -1.74 | 0.082 |
| Negative Mood × Disgust | 0.0514 |  | 0.0298 | 1.73 | 0.084 |
| Negative Mood × Sadness | -0.0500 |  | 0.0299 | -1.67 | 0.094 |
| Negative Mood × Fear | 0.1491 |  | 0.0297 | 5.03 | 0.000 |
|  |  |  |  |  |  |
| *C) Age group differences in mood-congruent versus mood-incongruent information processing* | | | | |  |
| Negative Mood × Younger Adults (Baseline: Neutrality) | -0.0587 |  | 0.0314 | -1.87 | 0.062 |
| Negative Mood × Older Adults (Baseline: Neutrality) | 0.0513 |  | 0.0422 | 1.22 | 0.224 |
| Negative Mood × Happiness × Younger Adults | 0.3287 |  | 0.0463 | 7.09 | 0.000 |
| Negative Mood × Happiness × Older Adults | -0.5497 |  | 0.0654 | -8.4 | 0.000 |
| Negative Mood × Anger × Younger Adults | 0.2393 |  | 0.0357 | 6.7 | 0.000 |
| Parameter | Estimate |  | Std. Error | *t*-value | *p*-value |
| Negative Mood × Anger × Older Adults | 0.0460 |  | 0.0459 | 1 | 0.316 |
| Negative Mood × Disgust × Younger Adults | 0.2511 |  | 0.0357 | 7.04 | 0.000 |
| Negative Mood × Disgust × Older Adults | -0.0853 |  | 0.0460 | -1.86 | 0.063 |
| Negative Mood × Sadness × Younger Adults | 0.1920 |  | 0.0358 | 5.37 | 0.000 |
| Negative Mood × Sadness × Older Adults | -0.0670 |  | 0.0461 | -1.45 | 0.146 |
| Negative Mood × Fear × Younger Adults | 0.2346 |  | 0.0356 | 6.6 | 0.000 |
| Negative Mood × Fear × Older Adults | -0.0040 |  | 0.0458 | -0.09 | 0.931 |
|  |  |  |  |  |  |
| *D) Temporal sequence in mood-congruent versus mood-incongruent information processing* | | | | |  |
| Stimulus Number (Baseline: Neutrality) | -0.0005 |  | 0.0004 | -1.38 | 0.168 |
| Happiness × Stimulus Number | -0.0026 |  | 0.0006 | -4.31 | 0.000 |
| Anger × Stimulus Number | -0.0024 |  | 0.0005 | -4.7 | 0.000 |
| Disgust × Stimulus Number | -0.0028 |  | 0.0005 | -5.51 | 0.000 |
| Sadness × Stimulus Number | -0.0015 |  | 0.0005 | -3.03 | 0.002 |
| Fear × Stimulus Number | -0.0016 |  | 0.0005 | -3.13 | 0.002 |
|  |  |  |  |  |  |
| Negative Mood × Stimulus Number (Baseline: Neutrality) | -0.0002 |  | 0.0007 | -0.36 | 0.719 |
| Negative Mood × Happiness × Stimulus Number | 0.0011 |  | 0.0010 | 1.06 | 0.288 |
| Negative Mood × Anger × Stimulus Number | 0.0005 |  | 0.0008 | 0.64 | 0.524 |
| Negative Mood × Disgust × Stimulus Number | 0.0013 |  | 0.0008 | 1.53 | 0.126 |
| Negative Mood × Sadness × Stimulus Number | 0.0009 |  | 0.0008 | 1.04 | 0.297 |
| Negative Mood × Fear × Stimulus Number | 0.0002 |  | 0.0008 | 0.29 | 0.768 |
|  |  |  |  |  |  |
| Recognition Ability | -10.9500 |  | 1.3970 | -7.83 | 0.000 |
|  |  |  |  |  |  |
|  | | | | |  |
| Random Intercept Face | Variance = 0.016; Std.Dev. = 0.127 | | | | |
| Random Intercept Participant | Variance = 1.624; Std.Dev. = 1.274 | | | | |
| AIC | 379146.2 |  |  |  |  |
| BIC | 379728.8 |  |  |  |  |
| Log-Likelihood | -189520.1 |  |  |  |  |
| Deviance | 379040.2 |  |  |  |  |

##### *Note*. Type of emotion was dummy coded with perception of neutral emotional expression as baseline. Session = grand mean centered session number; Session squared = squared grand mean centered session number; Age group of participant (younger, middle-aged, older adults) was effect coded; Negative mood was group mean centered by subtracting the average negative mood at each study session; Stimulus number = individually centered position of the stimulus in the sequence of stimuli within each session; Recognition ability = grand mean centered recognition ability per person. Faces and participants were treated as two crossed random effects.
